# Impact of Duration of Catheterization on the Success Rate of Trial Without Catheter in Acute Urinary Retention Due to Benign Prostatic Enlargement

**DOI:** 10.7759/cureus.42716

**Published:** 2023-07-31

**Authors:** Noman Ali Ghazanfar, Abdur Rasheed, Asad Ali Shah, Saad Akmal Bhatti, Hassan Sohail, Abid Farooq

**Affiliations:** 1 Urology, University College of Medicine and Dentistry/Social Security Teaching Hospital, Lahore, PAK; 2 Urology, Central Park Medical College and Teaching Hospital, Lahore, PAK; 3 Urology, Services Hospital Lahore, Lahore, PAK; 4 Urology, Blackpool Victoria NHS (National Health Services) Trust Hospital, Blackpool, GBR

**Keywords:** alpha blockers, bph, twoc, acute urinary retention, trial without catheter, urinary catheter

## Abstract

Background: The most common cause of acute urinary retention in men over 50 is benign prostate enlargement (BPE). Following the urethral catheterization, a trial without a catheter (TWOC) under the cover of alpha-blockers is given. The timing of TWOC varies from Day 3 to Day 7 of the retention episode. There is a need to study the improvement in the success rate of TWOC with the increasing number of days of catheterization.

Objective: To measure the success rate of TWOC in acute urinary retention due to benign prostatic enlargement with increasing days of catheterization.

Method: The study was conducted in Social Security Teaching Hospital Lahore. Patients who presented with acute urinary retention due to benign prostatic enlargement were catheterized and given alpha-blockers. The patients were divided into two groups, one group having TWOC after three days and the other having TWOC after seven days. The success rate of TWOC was calculated and compared in the two groups. All patients included in the study had the first episode of acute retention with a moderately enlarged prostate and no element of second pathology or neurological deficit.

Results: A total of 48 patients were included in the study, divided into two groups of 24 patients each. In the first group who underwent TWOC after seven days of catheterization, 15 out of 24 patients had successful TWOC with a success rate of 62.5%. In the second group of 24 patients, who had TWOC after three days of catheterization, only 11 patients had successful TWOC with a success rate of 45.8%.

Conclusion: There was a marked improvement in the success rate of TWOC with increasing days of catheterization after an acute retention episode, secondary to BPE.

## Introduction

Benign prostatic hyperplasia, i.e. benign prostate enlargement (BPE), is not uncommon in men over the age of 60 years. It may present with storage or voiding lower urinary tract symptoms, recurrent hematuria, or chronic retention with features of renal failure but acute urinary retention is still the most common presenting feature of BPE [1]. Acute urinary retention is defined as the sudden, painful inability to pass urine and is treated by urinary catheterization. This is followed by a trial without a catheter (TWOC) after two to seven days of a retention episode with or without the cover of alpha-blockers, provided there have been no previous similar episodes in the recent past.

There are several factors upon which the success of TWOC depends, including the age of the patient, the volume of the prostate, pre-void urinary volume, intravesical prostatic projection, presence or absence of alpha-blocker treatment, and others [2,3]. Among many other factors, one significant and less studied factor is the number of days of catheterization after the retention episode. Guidelines and textbooks suggest that TWOC can be given any time from Day 3 to Day 7 of the episode of retention.

The data and studies on the subject, however, are limited in Pakistan and there is a need to study the impact of the increasing number of days of catheterization on the success rate of TWOC with a vast potential of saving patients from unnecessary surgeries and treatments. There is a need to critically analyze the difference in the success rate of TWOC with increased days of catheterization. The purpose of this study was to measure, analyze, and compare the success rate of TWOC with increased days of catheterization, thus establishing whether it makes any difference in the patient outcomes with a potential to avoid transurethral resection of the prostate (TURP) [4-6] and its resultant complications in case of failure of TWOC.

## Materials and methods

After approval from the Ethical Review Committee of the Social Security Teaching Hospital Lahore, the patients who presented to the hospital with acute urinary retention were catheterized per urethra and enrolled in the study after providing their consent to participate. The patients included in the study were with moderate enlargement of the prostate on digital rectal examination with no features suggesting chronic retention, renal failure, or element of neurogenic bladder. They were given TWOC on Day 3 and Day 7 of the retention episode and were divided into two groups accordingly.

All patients were given alpha-blockers, 0.4 mg once daily, during the phase of catheterization. The results of TWOC were studied and compared in the two said groups. The failure of TWOC was defined as the painful inability to pass urine with a palpable bladder upon examination and post-void residual of greater than 150 ml on a departmental bladder scan. Following the success of TWOC, the patients continued on alpha-blocker therapy while those who failed were re-catheterized and managed further accordingly. The inclusion and exclusion criteria were as follows:

Inclusion criteria:

1) Males

2) Age 50-85

3) Patients with the first episode of retention

4) Moderately enlarged prostate (upper limit reachable upon digital rectal examination, 40 to 70 grams on ultrasound)

5) Patients who consent to participate.

Exclusion criteria:

1) Females

2) Patients with previous episodes of retention

3) Patients with chronic retention

4) Postoperative patients going into retention

5) Patients with known co-morbidities including neurogenic bladder

6) Co-existing pathologies apart from BPE

7) Patients with unsuitable profiles for the use of alpha-blockers.

## Results

**Figure 1:**
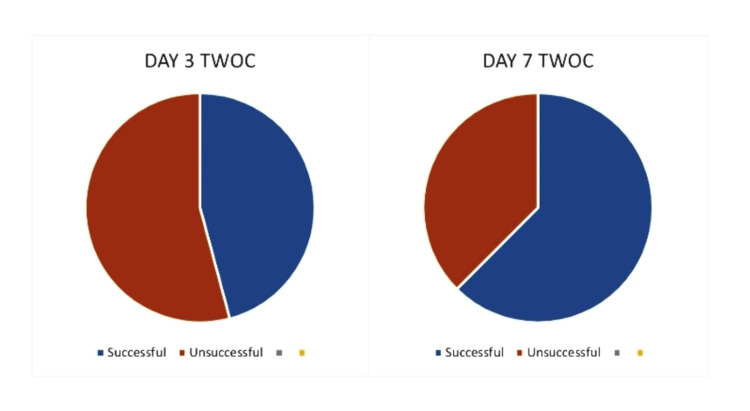
Comparison of the success rates of TWOC in two patient groups TWOC, trial without a catheter.

A total of 48 patients were included in the study, divided into two groups having 24 patients each. The mean age of the patient was 64.3 years ± 8.97. In the Day 7 TWOC group who had TWOC after seven days of catheterization, 15 out of 24 patients had successful TWOC with a success rate of 62.5%, whereas nine patients out of 24 failed their TWOC with a failure rate of 37.5%. In the Day 3 TWOC group who had TWOC after three days of catheterization, 11 out of 24 patients had successful TWOC with a success rate of 45.9%, whereas 13 patients out of 24 in this group failed their TWOC with a significant failure rate of 54.1% (Figure 1). In comparison, an additional success rate of 16.6% was achieved in the Day 7 TWOC group, which is quite significant (Table 1). This success rate also comes up with the benefit of lower rates of re-catheterization and refractory urinary retention. This ultimately led to lesser chances of surgical intervention in the form of TURP. TURP itself is a procedure that brings an array of potential complications and patient dissatisfaction (Table 2), which can be avoided with the success of TWOC and a prolonged catheterization of seven days after a retention episode.

**Table 1:** Comparison between two groups of TWOC with respective success rates TWOC, trial without a catheter.

TWOC group	Total no of patients	Patients with successful TWOC	Patients with unsuccessful TWOC	Success rate	Failure rate	Additional success rate compared with other group
Day 7	24	15	09	62.5%	37.5%	16.6%
Day 3	24	11	13	45.9%	54.1%	----

**Table 2:** TURP and its major complications TURP, transurethral resection of the prostate. Source: [7].

S. no	Parameter assessed after TURP surgery	Percentage of patients experiencing the complication
1	Patient dissatisfaction	16%
2	Failure rate	4.6%
3	Urinary incontinence	3%
4	Urinary tract infections	6%
5	Bladder neck contracture	7%
6	Transfusion rate	8%
7	Cardiovascular/thromboembolic complications	2%
8	Secondary procedure	5%

## Discussion

TWOC is an important critical step in the management of acute urinary retention due to benign prostatic enlargement. Successful TWOC is followed by medical management of the continuation of alpha-blocker treatment in most of the cases. Failure of TWOC is termed refractory urinary retention and is an indication of TURP or other surgical procedures [7]. The potential complications of TURP include urinary incontinence, permanent retrograde ejaculation, erectile dysfunction, infection, sepsis, TURP syndrome along with other complications associated with anesthesia. Any factor increasing the success rate of TWOC is not only critical for the treating urologist but also hugely beneficial for the patient as well. Although increasing days of catheterization increase morbidity [8], it also provides a significant increase in the success rate of TWOC.

Worldwide, different studies have been conducted analyzing different factors contributing to the success of TWOC in BPH patients including the age of the patient, the American Urological Association (AUA) score, and the presence or absence of alpha-blocker treatment. Some other studies have compared the effectiveness of different types of alpha-blockers in TWOC success and many other factors. Limited data and studies are available, analyzing the impact of the increased number of days of catheterization on the success rate of TWOC. The critics of prolonged catheterization suggest catheter removal after the standard three days, advocating increased morbidity with a prolonged catheterization of seven days due to the potential of urinary tract infections [9], hematuria, and limitation of daily activities and mobility. This can be avoided by aseptic techniques, the use of antibiotics in the susceptible groups [10], the use of leg bags and plug bags with lock-out valves [11], and multimodal management of catheters. In addition, there is a very small difference in complications of catheterization seen with additional four days of catheterization, whereas the same provides a significant improvement in the success rate of TWOC with a potential to avoid TURP surgery and its complications. In an era of minimal-invasive and non-invasive treatment strategies, our study has shown additional chances of success in TWOC with an increased duration of catheterization, but with standardizing the variability in other factors including the age of the patient, prostate volume, AUA score, and other factors.

The overall success rate of TWOC seen in a large worldwide survey published by British Journal of Urology was around 61% after a median of five days of catheterization [12], which is very similar to our study, while another study conducted in India showed that increasing number of days of catheterization does not make any significant difference to chances of success after TWOC [13], which is in contrast to our study where a significant difference of 16.6% was seen with seven days of catheterization and re-catheterization was significantly reduced. Significant controversy exists in the literature regarding days of catheterization, i.e. early versus late TWOC [14], with urologists having tangible arguments on both sides of the debate. Another study published in the Chinese Journal of Geriatrics showed a slightly better success rate of TWOC after a longer duration of catheterization similar to our study. We have shown with evidence in our study that longer catheterization of seven days does increase the chances of success with far less re-catheterization rate as compared to an early TWOC of three days, with a little difference in the side effects related to catheterization [15].

The limitations of our study however include small sample size, and variability in factors like prostate volume, AUA score, and intravesical prostatic projection, which affect the success rate of TWOC. We tried to minimize this by our appropriate inclusion and exclusion criteria with considerable standardization of these factors among patients included in our study; however, we plan to address these factors and their impact on the success of TWOC in our future studies.

## Conclusions

It is concluded that there is a significant increase in the success rate of TWOC after an event of urinary retention in BPE, with an increased duration of catheterization. Removal of the urinary catheter on Day 7 of the retention episode increases the chances of success significantly as compared to the removal of the catheter on Day 3, while the former does not significantly increase the chances of morbidity or catheter-related infections. Keeping the other parameters affecting the success rate of TWOC like prostate volume, AUA score, and intravesical prostatic projections as standard, a relatively longer period of catheterization can be critical in saving the patient from unnecessary surgical interventions like TURP and their resultant complications that occur in the case of failure of TWOC and re-catheterization. This proclaims seven days of catheterization being more favorable than three days of catheterization practice, after an acute urinary retention episode in BPE.
